# Decision-Model Estimation of the Age-Specific Disability Weight for Schistosomiasis Japonica: A Systematic Review of the Literature

**DOI:** 10.1371/journal.pntd.0000158

**Published:** 2008-03-05

**Authors:** Julia L. Finkelstein, Mark D. Schleinitz, Hélène Carabin, Stephen T. McGarvey

**Affiliations:** 1 Department of Nutrition, Harvard School of Public Health, Boston, Massachusetts, United States of America; 2 Department of Community Health, Brown University, Providence, Rhode Island, United States of America; 3 Department of Medicine, Brown University, Providence, Rhode Island, United States of America; 4 Department of Medicine, Rhode Island Hospital, Providence, Rhode Island, United States of America; 5 Department of Biostatistics and Epidemiology, University of Oklahoma Health Sciences Center, Oklahoma City, Oklahoma, United States of America; 6 International Health Institute, Brown University, Providence, Rhode Island, United States of America; Case Western Reserve University School of Medicine, United States of America

## Abstract

Schistosomiasis is among the most prevalent parasitic infections worldwide. However, current Global Burden of Disease (GBD) disability-adjusted life year estimates indicate that its population-level impact is negligible. Recent studies suggest that GBD methodologies may significantly underestimate the burden of parasitic diseases, including schistosomiasis. Furthermore, strain-specific disability weights have not been established for schistosomiasis, and the magnitude of human disease burden due to *Schistosoma japonicum* remains controversial. We used a decision model to quantify an alternative disability weight estimate of the burden of human disease due to *S. japonicum*. We reviewed *S. japonicum* morbidity data, and constructed decision trees for all infected persons and two age-specific strata, <15 years (y) and ≥15 y. We conducted stochastic and probabilistic sensitivity analyses for each model. Infection with *S. japonicum* was associated with an average disability weight of 0.132, with age-specific disability weights of 0.098 (<15 y) and 0.186 (≥15 y). Re-estimated disability weights were seven to 46 times greater than current GBD measures; no simulations produced disability weight estimates lower than 0.009. Nutritional morbidities had the greatest contribution to the *S. japonicum* disability weight in the <15 y model, whereas major organ pathologies were the most critical variables in the older age group. GBD disability weights for schistosomiasis urgently need to be revised, and species-specific disability weights should be established. Even a marginal increase in current estimates would result in a substantial rise in the estimated global burden of schistosomiasis, and have considerable implications for public health prioritization and resource allocation for schistosomiasis research, monitoring, and control.

## Author Summary

Schistosomiasis is a parasitic infection caused by a flatworm that disproportionately affects the world's poorest populations. Schistosomiasis is one of the most common infections worldwide, affecting over 207 million people in 76 countries. Current international estimates indicate that schistosomiasis has a minimal impact at the population level. This has contributed to its low prioritization in global health and subsequent resource allocation for disease control. However, recent studies indicate that these measures underestimate the extent of neglected tropical diseases, including schistosomiasis. Despite World Health Organization recommendations, the burden of schistosomiasis has not been re-examined in over a decade, and there are no established estimates for different types of schistosomiasis. The impact of symptoms associated with the Asian strain, *Schistosoma japonicum*, remains controversial. This study was conducted to provide an alternate measure of the burden of *S. japonicum*. We reviewed the literature and calculated a summary estimate for *S. japonicum* which was seven to 46 times greater than current measures for schistosomiasis. Findings suggest that current measures severely underestimate the extent of schistosomiasis, and urgently need to be revised. Further research is needed to examine the burden of schistosomiasis and other forgotten tropical diseases affecting the world's poorest people in endemic countries.

## Introduction

Schistosomiasis is one of the most prevalent parasitic infections worldwide. An estimated 779 million people are at risk for schistosomiasis, with 207 million infected in 76 countries and territories [1],[2]. Approximately 120 million people are symptomatic and 20 million have severe and debilitating disease [3]–[5]. Schistosomiasis accounts for 1.7 [6],[7] to 4.5 million disability-adjusted life years (DALYs) [8] lost each year worldwide, among the highest of all neglected tropical diseases.

Schistosomiasis japonica is caused by the trematode *Schistosoma japonicum*. Schistosome egg deposition in tissue and subsequent inflammatory immune response result in extensive clinical manifestations, including hepatomegaly, splenomegaly, and liver fibrosis [9]–[16], as well as “subtle” morbidities such as anemia, diarrhea, growth retardation, and cognitive deficits [10], [17]–[21]. Schistosomiasis japonica may be more pathogenic compared to other schistosomes affecting humans, due to comparatively higher egg production [22]. However, the burden of human disease due to *S. japonicum* infection is not well-established.

Global Burden of Disease (GBD) estimates indicate that the population-level impact of schistosomiasis is negligible. Schistosomiasis accounts for only 0.1% of the global burden of disease [23]. A major limitation of the GBD burden estimates for schistosomiasis is their restriction to the period of acute infection, excluding a number of chronic, severe, and debilitating morbidities, such as liver cirrhosis and cognitive deficits, which were included in disability estimations for other infections. As a result, age-specific GBD disability weights were estimated to be 0.005 for those <15 years (y) of age and 0.006 for those ≥15 y, on a scale from 0 (no impairment) to 1 (death) [8],[17],[19],[24]. Similar GBD weights have been assigned to relatively minor conditions such as facial vitiligo [8],[17],[19],[24]. In contrast, some studies [19],[24] have suggested that the actual burden of schistosomiasis is several-fold higher than current GBD estimates [8],[17],[19],[24]. For example, a recent systematic review and meta-analysis of all schistosome strains [19] and a community-based study of chronic schistosomiasis japonica in China [16] concluded that the GBD disability weights underestimate the extent of disability due to schistosomiasis; re-estimated disability weights for schistosomiasis were four to 30 times higher than current GBD measures [19], [23], [25]–[27].

Another limitation of the GBD study [23], [25]–[27] and subsequent schistosomiasis burden assessments [19],[24] was their estimation of disability weights for all three major schistosome species (*S. japonicum*, *S. mansoni* and *S. haematobium*) together, despite their distinct pathophysiology and associated morbidities. A recent community-based study in China using a standard quality of life measurement scale (EuroQol) suggested that species-specific estimation of disability weights for schistosomiasis is warranted, and that GBD values aggregated across all schistosome species may underestimate the disability associated with *S. japonicum* [16]. However, this study excluded nutritional and neurological morbidities, and may therefore still underestimate the burden of human disease due to *S. japonicum* infection.

The burden of schistosomiasis has not been re-examined in over a decade, despite three revisions to the GBD study [25]–[27] and a strong recommendation from the World Health Organization (WHO) [8]. Additionally, there is a lack of international consensus in disease burden assessment criteria, disability weights, and estimated burden for schistosomiasis. For example, in 2002, a WHO Technical Report recalculated the global burden of schistosomiasis at 4.5 million DALYs, and asserted that the previous estimate of 1.7 million DALYs lost to schistosomiasis (2001) “represents a serious underestimate and should be revised” [8]. However, the WHO continues to report the 1.7 million DALYs figure from 2001 [6],[7]. Further, there is a more than ten-fold difference between GBD and WHO estimates for schistosomiasis-related mortality, or 15,000 to 280,000 deaths per year [8],[23].

At present, no global species-specific burden assessment exists for schistosomiasis. The burden due to *S. japonicum* remains controversial and warrants further investigation. This study was conducted to quantify age-specific disability weight estimates for the burden of human disease due to *S. japonicum* infection using a decision model approach.

## Methods

### Literature Search Strategy

We conducted a structured literature search using MEDLINE electronic database to identify published studies from Jan 1, 1966 to May 1, 2007. A detailed summary of key words and search headings are provided in **Appendix S1**. Additional sources were identified from bibliographies of published studies, hand searches of scientific meeting abstracts, expert committee reports, and unpublished manuscripts and theses at Brown University, United States. We also solicited unpublished studies from known schistosomiasis japonica researchers *via* e-mail to minimize publication bias. These sources were retrieved, collected, indexed, and assessed for morbidity and disability outcome data.

**Figure 1:**
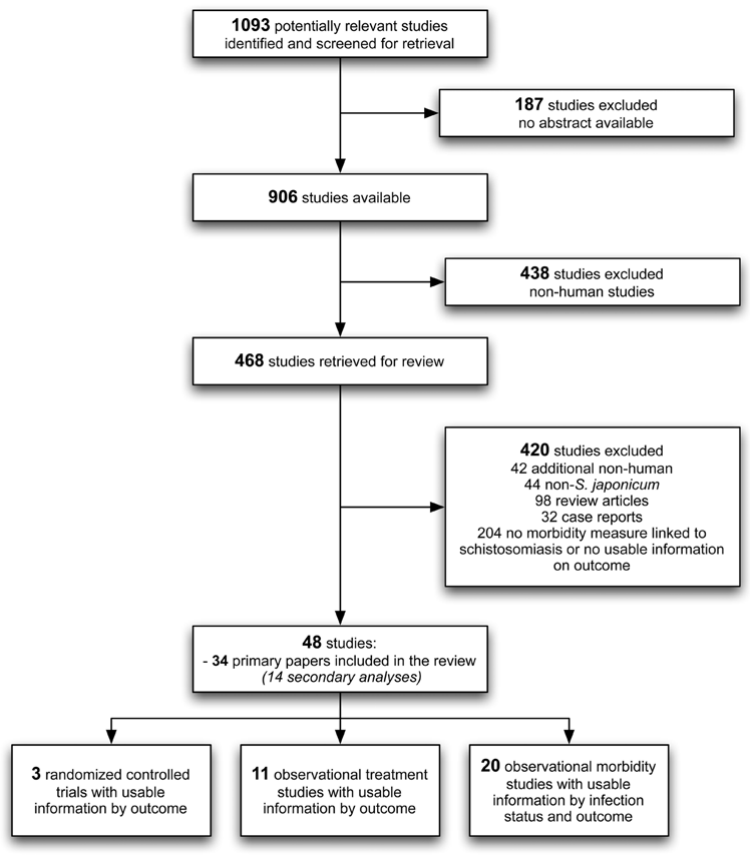
Literature search strategy. Shown is a diagrammatic representation of the retrieval strategy used for identifying and selecting studies for inclusion in the final analysis.

The initial inclusion criteria for this review were the availability of an abstract and a focus on human infection with schistosomiasis. The abstracts of all remaining studies were reviewed and the following inclusion criteria were applied: (i) focus on *S. japonicum*; (ii) human studies; (iii) experimental or observational (treatment, morbidity) study designs; and (iv) description of *S. japonicum* morbidity measures and their respective prevalences and/or disability weights (**Figure 1**). Additional information on publication date, author, location, population, study type, and relevant outcomes were recorded. Where available, we collected morbidity estimates based upon standardized clinical physiological data and WHO diagnostic criteria [28] such as grades I through III clinical classification for hepatic fibrosis and cirrhosis [29] or Hackett score ≥2 to define splenomegaly [30]. Although major organ pathologies such as hepatic and spleen morbidities have not been extensively studied in schistosomiasis, these morbidities were included as disability-related outcomes in the GBD disease burden assessment for other conditions. We included these morbidities in this assessment of disability-related outcomes in *S. japonicum*, based on our *a priori* hypothesis that they may impose a heavy burden on infected individuals.

### Model Structure

We identified a broad spectrum of morbidities associated with *S. japonicum,* namely: diarrhea, gastrointestinal bleeding, abdominal pain, hepatomegaly (mild/moderate, severe), hepatic fibrosis and cirrhosis (mild, moderate, severe), splenomegaly, cognitive deficits, stunting, wasting, anemia (mild, moderate, severe), central nervous system disease (i.e., including non-epilepsy neurological manifestations), and epilepsy.

Decision trees were used to systematically combine a large number of prevalence and disability data for *S. japonicum* morbidities [31]. The disability weight for schistosomiasis was estimated in three models to represent all ages, those aged <15 y and those aged ≥15 y, in order to facilitate comparison with the GBD study. For each model, the disability weight was estimated as:
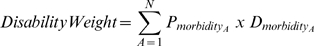
where *P_morbidity_A__* represents the probability of the A^th^ morbidity, *D_morbidity_A__* represents the disability weight associated with the A^th^ morbidity, and N is the total number of morbidities considered.

In general, we structured the decision trees to allow for all plausible combinations of morbidities, with the presence or absence of any single morbidity considered independently from other morbidities, with the exception of liver morbidity. Our model was therefore a series of successive binary branches, each depicting the presence or absence of a single morbidity. This means that although the probability of developing each morbidity was less than 1 by definition, the sum of all morbidity probabilities could exceed 1.

**Figure 2:**
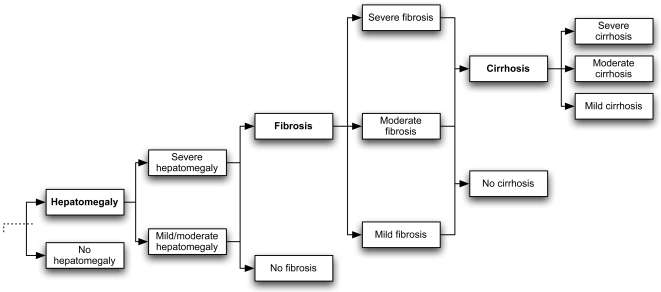
Schematic representation of the model. Shown is the branch of the model depicting liver pathology, which may or may not be present. If present, there may be hepatomegaly of varying degrees. Regardless of the degree of hepatomegaly, fibrosis may exist. Cirrhosis could only occur when fibrosis was present. Other comorbidities did not depend on the presence of other conditions.

Based on available hepatic pathophysiology data, we conservatively structured the model to restrict liver pathologies according to usual liver disease progression (i.e. hepatomegaly to fibrosis to cirrhosis), and allow for the co-occurrence of only hepatomegaly and fibrosis. This is because, as the disease progresses to more severe pathology, the associated disability weight also increases. Therefore, if a combination of several liver co-morbidities had been allowed, the disability weight for liver disease could be overestimated. **Figure 2** illustrates the branch of the decision tree for the liver pathologies.

### Model Inputs

#### Probabilities

Prevalences of *S. japonicum* morbidities were used as the measure of occurrence, instead of incidence rates, due to the unavailability of incidence estimates and to allow comparability with prior studies [19], [23], [25]–[27]. Since prevalence is proportional to the incidence multiplied by the duration of the morbidity, measures reflected in these model inputs represent a combination of the time spent in a morbidity state and its actual occurrence.

For each morbidity, the lowest prevalence estimate identified from the literature was used as the average value of a beta (β) distribution. The lowest estimates were systematically selected for the lowest values, in order to undertake a highly conservative approach and foster comparability of findings to the GBD study. The range of each morbidity prevalence estimate reported in the literature was used to represent the variance of the β distribution. The β distribution was selected because it satisfied the *a priori* requirements to restrict the probability estimate range between 0 and 1, and enabled calculation and summarization of available prevalence data and the level of uncertainty associated with these estimates [32].

#### Disability Weights

We examined the original and revised GBD assessments to identify disability weights for morbidities associated with *S. japonicum* [23], [25]–[27]. For example, although schistosome infection is the only sequela considered in the GBD assessment for schistosomiasis, morbidities such as diarrhea, anemia, cognitive deficits, and cirrhosis are available in burden evaluations of other parasitic diseases (e.g., ascariasis). We compiled these disability weight estimates, supplemented findings with data derived from literature sources, and consulted experts working on schistosomiasis japonica in countries where this disease is endemic (e.g., the Philippines and China) and from the United States, to confirm conservative proxy disability weight estimates. For each morbidity, an approach similar to the one described for the prevalence of each morbidity was used to represent the value and uncertainty of the disability weights.

### Base-Case Analyses

We calculated a baseline disability weight for each model using the most conservative mean estimates for disability weight inputs for all morbidities. We re-evaluated each model after systematically excluding each input to evaluate the independent contribution of each variable to the overall disability weight estimate. Model inputs were considered critical variables if they contributed more than 10% to the overall disability weight.

### Sensitivity Analyses

#### One-way

We performed deterministic one-way sensitivity analyses for each variable, for both probability and disability weight inputs, in order to assess the degree to which variation in each model input altered findings. We re-evaluated each model at the lower and upper bounds of the 95% confidence interval (CI) for each input, and observed subsequent modifications to the model disability weight. Model inputs were considered critical variables if alteration resulted in at least 10% change in the overall disability weight.

#### Multi-way

We performed multi-way deterministic sensitivity analyses to explore the most extreme values for disability weight estimates in overall and age-specific models. In the conservative and pessimistic scenarios, we simultaneously considered the respective lower and upper bounds of the 95% CIs for all probability and disability weight inputs, respectively.

#### Probabilistic

Deterministic analysis is typically limited in its consideration of a small number of model inputs with fixed values. Conversely, probabilistic analyses allow all inputs to vary simultaneously according to their distributions. We performed probabilistic sensitivity analyses with 5,000 Monte Carlo simulations for each of the three models. In each simulation, the value for each model input was selected at random from its specified distribution. In each case, we obtained a probability density distribution for schistosomiasis japonica disability weight. We summarized the results across all simulations by graphing the mean, standard deviation, and ranges of disability weights over 5,000 Monte Carlo analyses in each model [33].

## Results

### Model Inputs

The retrieval search strategy for study selection and details regarding study inclusion and exclusion in this analysis are diagrammatically represented in **Figure 1**. We identified 1093 potentially relevant studies from 1966 to May 1, 2007. We excluded 187 studies because no abstract was available. From the remaining 906 publications, 438 non-human studies were excluded, and 468 studies were retrieved for a detailed review. After careful examination of the publications, a total of 420 studies were excluded, including: 42 additional non-human studies, 44 non-*S. japonicum*, 32 case reports, 98 review articles, and 204 with no morbidity measure linked to schistosomiasis and/or no useable information on morbidity outcomes. A total of 48 studies met the inclusion criteria for this assessment, including 34 primary publications and 14 secondary papers from the same studies (**Figure 1**).

### Model Disability Weight

#### Base-Case Analyses

The average probability and disability weight values used in the base-case analyses are provided in **Table 1**. There were more publications investigating the occurrence of schistosomiasis-associated diarrhea, anemia, hepatomegaly, liver fibrosis, and splenomegaly, compared to publications which examined schistosomiasis-associated nutritional morbidities (e.g., wasting), cognitive function, cirrhosis, and neurological manifestations.

**Table 1:** Probability and disability weight estimates used in the base model to estimate the disability weight of *S. japonicum*

	*Probabilities* 1			*Disability weights* 1			*References*
	Overall	<15 years	≥15 years	Overall	<15 years	≥15 years	
Diarrhea	0.120	0.146	0.059	0.097	0.107	0.087	[13], [15], [19], [23], [38], [39], [43]–[45]
Gastrointestinal bleeding	0.070	0.070	0.070	0.010	0.010	0.010	[46]–[48],[49]
Abdominal pain	0.162	0.137	0.187	0.060	0.060	0.060	[13], [15], [45], [46], [48], [50]–[54]
Mild/moderate hepatomegaly	0.430	0.449	0.735	0.060	0.060	0.060	[12], [13], [15], [43], [46], [48], [50], [52], [53], [55]–[72]
Severe hepatomegaly	0.240	0.125	0.232	0.070	0.070	0.070	
Mild fibrosis	0.320	0.427	0.492	0.060	0.060	0.060	[15], [18], [43], [46], [57], [58], [61], [62], [70], [72]–[74]
Moderate fibrosis	0.130	0.121	0.604	0.060	0.060	0.060	
Severe fibrosis	0.060	0.023	0.265	0.070	0.070	0.070	
Mild cirrhosis	0.040	-	0.184	0.330	0.330	0.330	[23],[45],[46],[75]
Moderate cirrhosis	0.020	-	0.041	0.330	0.330	0.330	
Severe cirrhosis	0.020	-	0.020	0.330	0.330	0.330	
Splenomegaly	0.220	0.236	0.248	0.070	0.070	0.070	[13], [15], [43], [44], [46], [50], [55]–[58], [60], [61], [63]–[65],[67],[70],[72],[76]
Cognitive deficits	0.070	0.070	0.035	0.024	0.024	0.024	[23],[46],[77]
Stunting	0.100	0.290	0.100	0.017	0.024	-	[10], [18], [20], [21], [23], [46], [49], [50], [77]–[79], [80]–[82]
Wasting	0.095	0.235	0.070	0.030	0.053	-	
Mild anemia	0.140	0.491	0.165	0.000	0.000	0.000	[18],[20],[23],[46],[49],[54],[80],[83]
Moderate anemia	0.100	0.306	0.105	0.011	0.011	0.120	
Severe anemia	0.050	0.100	0.050	0.110	0.111	0.111	
CNS disease	0.026	-	0.026	0.070	0.070	0.070	[46],[84]
Epilepsy	0.021	-	0.021	0.125	0.099	0.150	[23],[46],[84]

1 ***Note:*** Probabilities represent prevalence estimates for morbidities in each age group. Disability weights ranged from 0 (no impairment) to 1 (death) [23]. Additional details on the determination of those estimates can be found in the Methods section.

*S. japonicum* was associated with an average disability weight of 0.130 in the overall model, with age-specific disability weights of 0.098 in the <15 y model and 0.198 for the ≥15 y model. In the all-ages model, major organ pathologies contributed to the largest proportion of the total disability weight, including probability and disability weight estimates for mild/moderate and severe hepatomegaly, mild fibrosis, mild cirrhosis, and splenomegaly (**Table 2**). The contributions of the different morbidities also varied by age. In the <15 y model, diarrhea, wasting and severe anemia also contributed to a large proportion of the *S. japonicum* disability weight, in addition to mild/moderate hepatomegaly and splenomegaly. In contrast, major organ pathologies were the most important variables in the older cohort model, including mild/moderate hepatomegaly, mild cirrhosis, and splenomegaly (**Table 2**).

**Table 2:** Proportion of model disability weight attributable to each morbidity

	Overall		<15		≥15	
	Contribution to model disability weight	% of total disability weight (0.130)	Contribution to model disability weight	% of total disability weight (0.098)	Contribution to model disability weight	% of total disability weight (0.186)
Diarrhea	0.010	8	0.014	14	0.004	2
Gastrointestinal bleeding	0.010	8	0.001	1	0.001	1
Abdominal pain	0.009	7	0.007	7	0.009	5
Mild/moderate hepatomegaly	0.014	11	0.018	18	0.023	12
Severe hepatomegaly	0.015	12	0.007	7	0.011	6
Mild fibrosis	0.014	11	0.008	8	0.005	3
Moderate fibrosis	0.006	5	0.003	3	0.015	8
Severe fibrosis	0.003	2	0.001	1	0.010	5
Mild cirrhosis	0.013	10	0.000	0	0.054	27
Moderate cirrhosis	0.006	5	0.000	0	0.013	7
Severe cirrhosis	0.006	5	0.000	0	0.006	3
Splenomegaly	0.014	11	0.015	15	0.014	7
Cognitive deficits	0.001	1	0.002	2	0.001	1
Stunting	0.001	1	0.006	6	0.000	0
Wasting	0.002	2	0.011	11	0.000	0
Mild anemia	0.000	0	0.000	0	0.000	0
Moderate anemia	0.001	1	0.002	2	0.001	1
Severe anemia	0.005	4	0.010	10	0.005	3
CNS disease	0.002	2	0.000	0	0.001	1
Epilepsy	0.002	2	0.000	0	0.003	2

### Sensitivity Analyses

#### One-way

**Figure 3:**
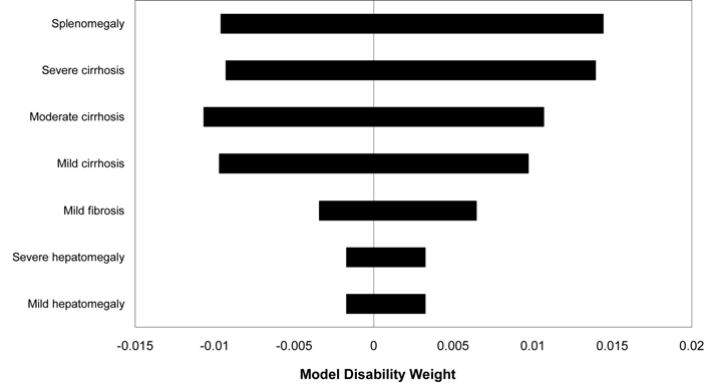
One-way sensitivity analysis. The horizontal bars depict the effect of re-evaluating the disability weight (shown on the X-axis) after changing the value of the specified parameter from the low to the high end of its range. The number of parameters that resulted in the greatest variation are shown.

The overall model was sensitive to probability estimates for mild/moderate and severe hepatomegaly, moderate and severe cirrhosis, and splenomegaly; and disability weight estimates for mild/moderate and severe hepatomegaly and mild fibrosis (**Figure 3**). As expected, the one-way sensitivity analyses varied by age. The <15 y model was most sensitive to probability and disability estimates for mild/moderate hepatomegaly and splenomegaly and to additional disability weight estimates for wasting and severe anemia. The ≥15 y model was also sensitive to probability and disability estimates for mild/moderate hepatomegaly, mild cirrhosis, and splenomegaly, and to the additional disability weight for moderate fibrosis.

#### Multi-way

Considering the most conservative estimate for each variable simultaneously resulted in a disability weight of 0.047 in the overall model, with age-specific estimates of 0.033 in the <15 y model, and 0.091 in the ≥15 y model. In the pessimistic scenario, we observed a maximum overall disability weight of 0.228, which ranged from 0.194 (<15 y) to 0.305 (≥15 y) in age-specific cohort analyses.

#### Probabilistic

**Figure 4:**
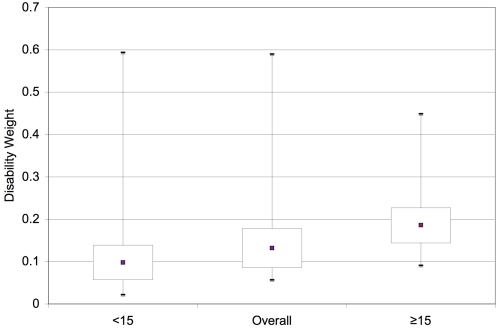
Probabilistic sensitivity analyses. Boxplots depicting the results of 5,000 simulation Monte Carlo analysis for each age-group model. Boxes represent the median, 25^th^ and 75^th^ percentiles, and error bars extend to the 2.5^th^ and 97.5^th^ percentile. Means are depicted by the circles.

Findings from probabilistic sensitivity analyses confirmed the magnitude and robustness of estimates from deterministic analyses. In the overall model, the average disability weight was 0.132 over 5,000 Monte Carlo simulations, with age-specific estimates of 0.098 (<15 y) and 0.186 (≥15 y). No simulations produced disability weight estimates lower than 0.009, and only 2.5% of simulations in the <15 y model were less than 0.044. Median values were 0.124 (2.5–97.5%: 0.080–0.232) for all ages, with age-specific estimates ranging from 0.092 (<15 y; 2.5–97.5%: 0.044–0.174) to 0.180 (≥15 y; 2.5–97.5%: 0.123–0.287) (**Figure 4**).

## Discussion

Our findings indicate that age-specific disability weights of 0.098 to 0.186 would be a more appropriate estimate for the burden of human disease due to *S. japonicum* infection. It is noteworthy that even the most conservative estimates were seven times greater than current GBD disability weights for schistosomiasis (**Table 3** **)** [23], [25]–[27].

**Table 3:** Disability weight estimates from review studies of all schistosome strains and schistosomiasis japonica

	*<15 years*	≥*15 years*	*Overall*	*Strains*	*Ratio vs. GBD*
**Global Burden of Disease** [23]	0.005	0.006	-	All	-
**King ** ***et al.*** **, 2005** [19]	-	-	0.020–0.150	All	4–30 : 1
**Jia ** ***et al.*** **, 2007** [16]	0.095^1^	0.159–0.246^2^	0.191	S. japonicum	19–27 : 1
**Current analysis**	0.098	0.186	0.130	S. japonicum	20–33 : 1^3^

1 This study excluded individuals <5 years (5–14 y).

2 This refers to the estimated disability weights for three age groups, namely: 15–44 y (0.159), 45–59 y (0.207), and ≥60 y (0.246).

3 Considering the lower and upper bounds of the confidence intervals for these estimates, the ratio vs. GBD estimates ranges from 7 to 46.

Findings in this study are consistent with King *et al.*'s [19] meta-analysis of disability-related outcomes in all strains of schistosomiasis. Minimum re-estimated disability weights in our assessment of 0.044 (<15 y) and 0.123 (≥15 y) were similar in magnitude to King *et al*'s estimates of 0.02 to 0.15, although our median values and upper estimates were considerably higher. This is concordant with the assertion that *S. japonicum* is more pathogenic than other schistosomes [22]. Our disability weight estimates are also consistent with findings from a community-based study in China that focused on chronic schistosomiasis japonica, reporting an overall disability weight of 0.191 and age-specific estimates of 0.095 (5–14 y) and 0.159 (15–44 y) [16]. Current findings and burden re-assessments by King and colleagues and Jia and colleagues are in contrast to two earlier reviews that suggested a minimal public health impact of schistosomiasis [34],[35].

There are several differences in King *et al.*'s meta-analysis of all schistosomes [19] and the China study focusing on chronic infections due to *S. japonicum* [16] compared to our analysis, particularly regarding the selection of schistosomiasis-related health conditions. In contrast to King *et al.*'s [19] assessment, we accounted for hepato-splenic pathologies due to *S. japonicum* infection, and identified them as critical disability-related outcomes, as did the Chinese study [16]. The exclusions of organ pathologies in King *et al.*'s assessment [19] and nutritional morbidities in the China study [16] may contribute to an underestimation of disability weights, relative to our findings. We also excluded work and school performance outcomes in our assessment, due to the limited availability of such studies in *S. japonicum* and a lack of objective standardized measures for these outcomes. Inclusion of these functional outcomes in future studies is expected to further increase estimated disability weights for *S. japonicum*.

There are several study limitations. Few incidence studies were available, and results are based on review of only 34 primary papers (48 total) with usable outcome information. Due to the nature of this analysis, which is to describe the natural history of acute and chronic *S. japonicum* infection, we decided not to formally score each study based on the quality of the information included. Hence, we assumed that every report represented the best available information for the specified population and outcomes at the time the study was undertaken [31]. Our analysis was limited by the research designs and topical foci of earlier studies. We did not include the entire range of possible morbidities and disabilities due to *S. japonicum* infection. We also excluded mortality-related outcomes and did not account for treatment status, co-morbidities with other infectious or non-infectious diseases, infection intensity, re-infection, or disease progression in order to facilitate comparability of findings to the GBD assessment [23], [25]–[27]. There is a possibility of publication bias in every review. To limit this, we solicited both published and unpublished manuscripts and consulted with experts in order to limit publication bias, and intentionally biased our model estimates to the lower ranges for each model parameter, in order to obtain conservative estimates. In addition, when more than one publication described the frequency of occurrence of a specific morbidity associated with schistosomiasis, the most conservative estimate was included in the assessment. These exclusions and limitations in available studies would likely lead to an underestimation of re-estimated model disability weights.

In schistosomiasis-endemic regions, polyparasitism, malnutrition, and other infectious diseases are ubiquitous. Therefore, it is important to interpret findings in the context of concurrent infections. For example, some disability-related outcomes, including anemia and diarrhea, may be due to the presence of other parasitic infections. Although polyparasitism may have contributed to an over-estimation of disability weights attributable to *S. japonicum*, this is unlikely since we used highly conservative estimates in this analysis. Recent studies have challenged the ability of experts to quantify patient-centered disability, particularly in chronic conditions characterized by low mortality [17],[19],[36],[37] and extensive co-morbidities [19],[38],[39]. However, the extent of the disparity between expert opinion and patient's experience is unknown. Expert panel methodologies may also underestimate the disability-related impact of nutritional morbidities, in favor of more standardized and observable organ pathologies. Therefore, the fundamental reliance on GBD disability weights in this assessment also represents an important study limitation.

Decision model estimation is a useful analytic method for conducting disease burden assessments; however, deterministic and probabilistic sensitivity analyses are exploratory, rather than explanatory. As a result, decision model estimation does not facilitate evaluation of the statistical significance or robustness of findings, which represents a study limitation. The β-distribution was selected for this analysis based on its satisfaction of *a priori* requirements to restrict the estimate ranges from 0 and 1, calculation and summarization of data, and evaluation of the level of estimate uncertainty [32]. Similar to other distributions, the β-distribution is influenced by the quality, availability, and level of skewness in model inputs. Therefore, availability and quality of epidemiological and disease burden data remain study limitations.

Further research is needed to examine the broad range of morbidities associated with schistosomiasis, particularly on methods to parse attributable causation of specific infections in the context of polyparasitism and other comorbid conditions. Additionally, the separation between specific causes of disease from associated morbidities, and the exclusion of selected conditions partially attributable to other infections in the GBD assessment may contribute to an underestimation of disability weights for schistosomiasis.

Lastly, as a zoonotic disease, schistosomiasis japonica is also a veterinary and agricultural public health issue. Future interdisciplinary research should consider the direct impact on human health and the indirect impact on animal health and economic productivity. Methods described by Budke *et al.* [40] and Carabin *et al*. [41] could also be utilized to broaden *S. japonicum* burden assessment to incorporate cost-effectiveness measures [40]–[42].

In conclusion, a minimum disability weight of 0.033 to 0.091 would be a more accurate estimate of disability due to *S. japonicum*. GBD methodologies underestimate the burden of disease attributable to *S. japonicum*, and hence they should be revised. Even a minimal increase in current estimates would result in a substantial rise in the estimated global burden of schistosomiasis, and have considerable implications for public health prioritization, health policy, and resource allocation for research, monitoring, and control.

## Supporting Information

Alternative Language Abstract S1 Translation of the abstract into Chinese by Ruilan Wei.
(0.14 MB PDF)

Alternative Language Abstract S2 Translation of the abstract into French by Hélène Carabin.
(0.02 MB DOC)

Alternative Langage Abstract S3 Translation of the abstract into Spanish by Susie Welty and Elena Gibbons.
(0.03 MB DOC)

Appendix S1 Key words for search strategy used to review the literature on *S. japonicum* for Medline (1966–2007).
(0.06 MB DOC)
