# Supplementary material for: Decision-Model Estimation of the Age-Specific Disability Weight for Schistosomiasis Japonica: A Systematic Review of the Literature
Source: PLoS Negl Trop Dis. 2008 Mar 5;2(3):e158. doi: 10.1371/journal.pntd.0000158 (PMC2254314; doi:10.1371/journal.pntd.0000158)
Supplement: Alternative Language Abstract S1 — Translation of the abstract into Chinese by Ruilan Wei. (0.14 MB PDF) [file pntd.0000158.s002.pdf]

应用决策模型估测日本血吸虫病的年龄特异性失能权重

血吸虫病是全球最流行的寄生虫感染疾病之一。然而，从目前使用的全球疾病负担（GBD）伤残调整健康生命年估测值来看，血吸虫病对人类健康的影响可以忽略不计。最近的一些研究表明这种GBD方法有可能低估了包括血吸虫病在内的寄生虫疾病的负担。另外，对于血吸虫病来讲，目前还没有品种特异性的失能权重数据。关于日本血吸虫病对人类疾病负担的力度大小没有统一的结论。我们应用决策模型定量估测由于日本血吸虫病而引起的人类疾病的失能权重。我们综述了日本血吸虫病的疾病数据，建立了所有被感染人群以及两个年龄段被感染人群（小于15岁和等于大于15岁）的决策树。对每个模型我们都进行了随机的和概率的敏感性分析。结果表明，日本血吸虫病感染的平均失能权重为0.132，年龄

小于15岁的人群为0.098，大于等于15岁的人群为0.186。我们的估测值大于GBD估测值7-46倍。模拟结果表明没有任何估测值低于0.009。对于小于15岁的~~人群~~感染人群，营养性疾病是导致由于日本血吸虫感染而引起的失能权重最重要的因素。而对于等于大于15岁的人群，~~器~~器官病变是最重要的原因。GBD失能权重急需改进，同时需要建立品种特异性的失能权重。既使一个很小的失能权重的提高，也会引起血吸虫病的全球负担估测的大大的提高。这对于公共健康的重要性排序，血吸虫疾病的研究和监控的资源分配，均具有重要意义。
