# Supplementary material for: Decision-Model Estimation of the Age-Specific Disability Weight for Schistosomiasis Japonica: A Systematic Review of the Literature
Source: PLoS Negl Trop Dis. 2008 Mar 5;2(3):e158. doi: 10.1371/journal.pntd.0000158 (PMC2254314; doi:10.1371/journal.pntd.0000158)
Supplement: Alternative Language Abstract S2 — Translation of the abstract into French by Hélène Carabin. (0.02 MB DOC) [file pntd.0000158.s003.doc]

***Decision-model Estimation of the Age-specific Disability Weight for Schistosomiasis japonica***

**Abstract:**

La bilharziase est l’une des maladies les plus prévalente dans le monde. Cependant, les estimations de la charge de morbidité mondiale (CMM) associés avec cette infection suggèrent que son impact est négligeable. Des études récentes semblent indiquer que les méthodes utilisées pour calculer la CMM sous-estiment considérablement le fardeau des maladies parasitaires, et tout particulièrement celui de la bilharziase. De plus, il n’existe pas de poids d’incapacité spécifique à chaque espèce de bilharziase et le fardeau associé avec *Schistosoma japonicum* demeure controversé. Nous avons utilisé la méthode des arbres de décision pour revoir les poids d’incapacité associés avec *S. japonicum*. Nous avons revu la littérature sur la fréquence des morbidités associées avec *S. japonicum* et avons organisé les résultats avec les arbres de décision pour trois groupes soit: tous les âges, ceux âgés de <15 ans et ceux âgés de ≥15 ans. Nous avons également mené des analyses de sensitivités avec des modèles probabilistes et stochastiques. L’infection avec *S. japonicum* est associée avec une moyenne de poids d’incapacité de 0.132 pour tous les âges et de 0.098 et 0.186 pour les <15 ans et ≥15 ans, respectivement. Ces nouvelles estimations sont de sept à 46 supérieures à celles du projet de la CMM. Aucune simulation n’était inférieure à 0.009. Chez les <15 ans, la malnutrition contribuait le plus au poids d’incapacité alors qu’il s’agissait de la pathologie des organes majeurs chez les ≥15 ans. La Il est impératif que la CMM pour la bilharziase soit révisée et que les poids d’incapacité spécifiques à chaque espèce soient estimés. Une augmentation même marginale de l’estimation actuelle du poids d’incapacité associé avec la bilharziase augmenterait considérablement sa contribution à la CMM et pourrait donc avoir des conséquences importantes en termes de prises de décision concernant la recherche, la surveillance, et la mise en place de mesures de contrôle de cette infection.
