# Supplementary material for: Decision-Model Estimation of the Age-Specific Disability Weight for Schistosomiasis Japonica: A Systematic Review of the Literature
Source: PLoS Negl Trop Dis. 2008 Mar 5;2(3):e158. doi: 10.1371/journal.pntd.0000158 (PMC2254314; doi:10.1371/journal.pntd.0000158)
Supplement: Alternative Langage Abstract S3 — Translation of the abstract into Spanish by Susie Welty and Elena Gibbons. (0.03 MB DOC) [file pntd.0000158.s001.doc]

***Decision-model Estimation of the Age-specific Disability Weight for Schistosomiasis japonica***

**Abstract:**

La esquistosomiasis está entre las infecciones parásitas más frecuentes por todo el mundo. Sin embargo, les estimaciones de la carga mundial de morbilidad (CMM) para la esquistosomiasis indican que su impacto del población-nivel es insignificante. Los estudios recientes sugieren que las metodologías de CMM puedan subestimar perceptiblemente la carga de enfermedades parásitas, incluyendo la esquistosomiasis. Además, los pesos species-específicos de la inhabilidad no se han establecido para la esquistosomiasis, y la magnitud de la carga humana de la enfermedad debido al *Schistosoma japonicum* sigue siendo polémica. Utilizamos un modelo de la decisión para cuantificar una estimación alternativa del peso de la inhabilidad de la carga de la enfermedad humana debido al *S. japonicum* Repasamos datos de la morbosidad del *S. japonicum*, y los árboles de decisión para todas las personas infectadas y dos estratos específicos a la edad, <15 años y ≥15 años. Condujimos los análisis estocásticos y probabilisticos de la sensibilidad para cada modelo. La infección con *S. japonicum* fue asociada a un peso medio de la inhabilidad de 0.132, con los pesos específicos a la edad de la inhabilidad de 0.098 (<15) y 0.186 (≥15). Los pesos re-estimados de la inhabilidad eran siete a 46 veces mayores que las medidas actuales de la CMM. Ningunas simulaciones produjeron las estimaciones más bajo de 0.009 del peso de la inhabilidad. Las morbosidades alimenticias tenían la contribución más grande al peso de la inhabilidad del *S. japonicum* en el modelo de <15 mientras que es principal las patologías del órgano eran las variables más críticas de la categoría de la edad más avanzada. Los pesos de la inhabilidad de CMM para la esquistosomiasis necesitan ser revisados urgentemente, y los pesos species-específicos de la inhabilidad necesitan ser establecidos. Incluso un aumento marginal en estimaciones actuales subida substancial de la carga global estimada de la esquistosomiasis, y tiene implicaciones considerables para la prioritización de la salud pública y asignación de recurso para la investigación, la supervisión, y el control de la esquistosomiasis.
