# Supplementary material for: Decision-Model Estimation of the Age-Specific Disability Weight for Schistosomiasis Japonica: A Systematic Review of the Literature
Source: PLoS Negl Trop Dis. 2008 Mar 5;2(3):e158. doi: 10.1371/journal.pntd.0000158 (PMC2254314; doi:10.1371/journal.pntd.0000158)
Supplement: Appendix S1 — Key words for search strategy used to review the literature on S. japonicum for Medline (1966–2007). (0.06 MB DOC) [file pntd.0000158.s004.doc]

**Appendix**

| **Appendix S1. Panel: Key words for search strategy used to review the literature on *S. japonicum* for Medline (1966-2007)** |
| --- |
| “schistosom* japonica*” combined with the following key terms, specified below:  “abdominal pain”, “anemia”, “bleeding”, “body fat”, “burden”, “burden assessment”, “cerebral”, “child development”, “central nervous system”, “cognition”, “cirrhosis”, “diarrhea”, “disability”, “growth”, “height”, “hepatic fibrosis”, “hepatomegaly”, “hepato-splenic”, “liver fibrosis”, “memory”, “morbidity”, “neurological”, “nutrition”, “nutritional status”, “prevalence”, “disability adjusted life year”, “DALY”, “quality adjusted life year”, “skin-fold thickness”, “spleen”, “splenomegaly”, “stool”, and “weight” |
